# Research on comprehensive detection and visualize of hidden cavity goaf

**DOI:** 10.1038/s41598-022-26680-3

**Published:** 2022-12-24

**Authors:** Bo Cao, Jian Wang, Han Du, Yabin Tao, Guangwei Liu

**Affiliations:** 1 College of Mining, Liaoning Technical University, Fuxin, 123000 China; 2 Department of Hydraulic Engineering, State Key Laboratory of Hydroscience and Engineering, Tsinghua University, Beijing, 100084 China; 3 School of Energy and Mining Engineering, China University of Mining and Technology (Beijing), Beijing, 100083 China

**Keywords:** Natural hazards, Civil engineering

## Abstract

At present, the research on goaf at home and abroad mainly focuses on four aspects: detection technology, stability evaluation technology, governance technology and quality control technology. The most important of the above four aspects is goaf detection technology. In order to ensure the accuracy and precision of exploration, many geophysical methods and high-density geological drilling are usually used for exploration. In case of complex terrain, this method will increase the workload rapidly, and can not achieve a good balance between exploration cost and exploration quality. Goaf exploration methods are still in the development stage, and each geophysical exploration method has its limitations. This study makes full use of the existing detection technology to detect the complex mined-out area of East Open-pit Mine, 9 inferred mined-out areas and 9 suspected mined-out areas were found by using 3D seismic exploration method, transient electromagnetic method is used to delineate 223 abnormal areas at different elevations within the exploration range. 58 drilling holes are arranged in the suspected mined-out area of East Open-pit Mine. Combined with geological software, 3D model map of mined-out area is drawn, and the causes of formation of mined-out area are classified and analyzed. Using 3D laser scanning technology to study the visualization of hidden mined-out areas, the hidden mined-out areas are divided into three types through visualization research, and its formation mechanism is analyzed. It can be applied to detection of open-pit mines which have small underground coal mines and many mined-out areas with complex geometric shapes and has great significance to the proposal of stability treatment scheme of mined-out area. The novelty of this study is prove the area, shape, roof thickness and height of the mined-out area by using joint detection method and the hidden mined-out area is visualized by 3D laser scanning technology.

## Introduction

China is still a big country that consumes coal resources^1^. However, China's coal is mainly mined by underground coal mines^2^. Generally speaking, the exploitation of open-pit coal resources still accounts for a small proportion. This means that the underground coal mining will inevitably lead to the enlargement of the goaf, the enlargement of the cavity of the goaf, the enlargement of the number of the goaf and the increase of the exposure time of the goaf^3^. It will definitely cause the safety accidents in the mined-out area, so the stability of mined-out area is related to whether the mining enterprises can work normally and safely^4^.

Goaf detection technology has an important application in finding out the cavity of goaf^5^. With the maturity of goaf detection technology, computer technology and the introduction of various mathematical models, the stability research and disaster control of coal mine goaf show a situation of blooming flowers^6^. A large number of advanced equipment replace the traditional goaf detection technology. With the development of industrial technology in China, the detection technology of mining area has been improved continuously^7^, and there is a qualitative leap from the most primitive drilling exploration to various advanced physical exploration technologies nowadays^8^. Among them, the physical exploration technology is mainly transient electromagnetic method^9^, ground-penetrating radar detection method^10^, high-density resistivity method^11^, seismic reflection wave method^12^ and three-dimensional laser scanning technology^13^. It can accurately detect the cavity in the goaf in three dimensions, and then draw the model of the cavity in the computer software. Judging the failure mode and characteristics of roof from the mechanical distribution characteristics. Followed by the introduction of mathematical model, the prediction and evaluation model of goaf is established on the basis of gray analysis-correlation degree. These theories greatly enrich the mechanical model of goaf stability and stability control theory. Among them, the physical exploration technologies are mainly transient electromagnetic method^14^, ground penetrating radar detection method^15^, and the high density resistivity method^16^.

Cheng et al.^17^ have done a lot of work in advance detection of roadway. Using transient electromagnetic method technology, the physical space model expression method of secondary magnetic field (eddy current field) of transient electromagnetic method is given, and the concept of stratification excitation of magnetic couple source is put forward. Yu et al.^18^ studied the device configuration of transient electromagnetic method, and on this basis, put forward the data preprocessing technology of transient electromagnetic method, constructed the TEM information inversion system, and achieved good test results. Gui et al.^19^ took water inrush from coal mine goaf as the research object, combined with wavelet theory, identified and suppressed noise and other external interference factors, and detected water-accumulated goaf in different geology on the spot. Through practical application on the spot, the detection effect of Youyi electromagnetic method was good from the theoretical and technical aspects. On the basis of TEM response theory, Qi et al.^20^ established mathematical models by writing 3D finite element program to describe different models and parameters, and compared and analyzed different models and parameters, so as to obtain the attenuation law of induced signals in coal and rock media and other media, and the interference characteristics of metal media on TEM. Ground penetrating radar detection method (GPR technology) is to use high-frequency electromagnetic waves to send high-frequency electromagnetic waves into the ground in the form of short pulses and broad bands, which are reflected by underground rock strata or coal seams. With the help of computer processing technology, the structural characteristics of coal and rock masses can be obtained. Giannopoulos et al.^21^ analyzed and compared several electromagnetic waves on the basis of GPR technology theory, and put forward a new wave velocity processing method, that is, rigid interface reflection coefficient method. With the help of field drilling experiments, the technology and algorithm were tested in the field, and the processing method with high precision and strong operability was realized. Turkington et al.^22^ have studied and applied GPR technology for its wide applicability and high precision in shallow strata. The results show that GPR technology is completely feasible in shallow strata, and the obtained geological body information and bad geological body information are completely reliable. Xu et al.^23^ explored and studied the grouting behind the tunnel wall in the process of tunnel shield, and obtained the electromagnetic wave propagation characteristics and laws of grouting body through indoor experiments. On the research results, the distribution characteristics of grouting body were accurately identified by using the calculation results of GPR data, and the abnormal parts in grouting body could be identified according to the GPR images and calculation results. Peng et al.^24^ explored and studied the grouting behind the tunnel wall in the process of tunnel shield, and obtained the electromagnetic wave propagation characteristics and laws of the grouting body through indoor experiments. On the research results, the distribution characteristics of the grouting body were accurately identified by using the calculation results of GPR data, and the abnormal parts in the grouting body could be identified according to the GPR images and calculation results. With the maturity of computer technology, visualization of high-density resistivity method and computer image processing technology, the application of high-density resistivity method has become extensive with the processing technology of computer embedded algorithm. Lghoul et al.^25^ used high-density resistivity method to detect an iron ore tailings pond, and analyse the suspected seepage channels, and found out many weak areas of dam structure. During several tests, the electrical phase response structure diagram of the whole tailings dam was obtained. Some scholars have also done a lot of work on slope instability and failure, obtained a large number of geoelectric models about slope instability and failure, and conducted forward modeling and inversion simulation. From the inversion results, the inversion effect is better than the forward modeling effect. On this technology, the indoor resistivity experiment of soil-rock mixture was carried out. According to the resistivity inversion results, the threshold value of rock and soil instability and failure is established, and the early warning model of slope instability and failure is established based on the image comparison algorithm.

The significance study justification of this study lies in the following two points: First, since the existence of coal seams is not an ideal spatial three-dimensional body, and their geometric boundaries and three-dimensional spatial models are not particularly regular, ordinary measurements simply cannot be accurately detected, and the errors in the data obtained are large. Mines cannot take suitable ways to manage these mining cavity problems based on this information, and it is likely to bring serious safety problems to the subsequent production of the mine. Therefore, mines need to use advanced instruments for detection, so as to obtain an accurate model of the cavity body of the mining area. Secondlly, The use of one kind of equipment or instrument often can not achieve the detection effect of underground mining area, often resulting in unnecessary waste of human resources, so the use of multi-technology joint physical exploration method can effectively compensate for the shortcomings of a single instrument. In this study, the extraction zone in the study area is processed and the cavity model of the extraction zone is plotted in the computer based on the detection results. Finally, goaf exploration methods are still in the development stage, and each geophysical exploration method has its limitations. Making full use of the existing detection technology and combining with the general situation of specific projects to detect the complex goaf clearly, and making it visible by using 3D laser scanning, is of great significance to the proposal of the stability treatment scheme of goaf.

Although there are more research results on the monitoring of the mining area, and the results have been achieved, and the field engineering application effect is also good, but the actual engineering still faces more practical engineering problems. With the increase of the coal mining range in our selected study area, the safety problem of the mining area is prominent, and if a disaster occurs, it will certainly cause a series of chain disaster phenomena, and the difficulty of its safety control will be further increased. In view of the complex situation of underground goaf in the East open-pit mine, one kind of equipment or instrument often cannot achieve the detection effect of underground goaf, which often causes unnecessary waste of human resources. Therefore, for the detection of underground goaf in the East open-pit mine, joint geophysical exploration technology, namely joint physical exploration technology, is adopted. They are transient electromagnetic method, high density resistivity method, seismic reflection wave method and 3D laser scanning technology. Multi-technique combined geophysical exploration method can effectively make up the deficiency of single instrument. Preliminary use high-density three-dimensional seismic method, transient electromagnetic method, high density resistivity method to detect the approximate location of the underground mined-out area, and then find out by geological drilling method research area distribution and buried depth of goaf, the final distribution in goaf and buried depth to determine the scope of the case, then the 3 d laser scanning technology, More than 50 mined-out areas in east open-pit mine are treated and the relevant model results are obtained, which provide guarantee and reference information for the stability of mined-out areas.

## Engineering background and geological setting

### EngineeSring geological conditions

**Figure 1:**
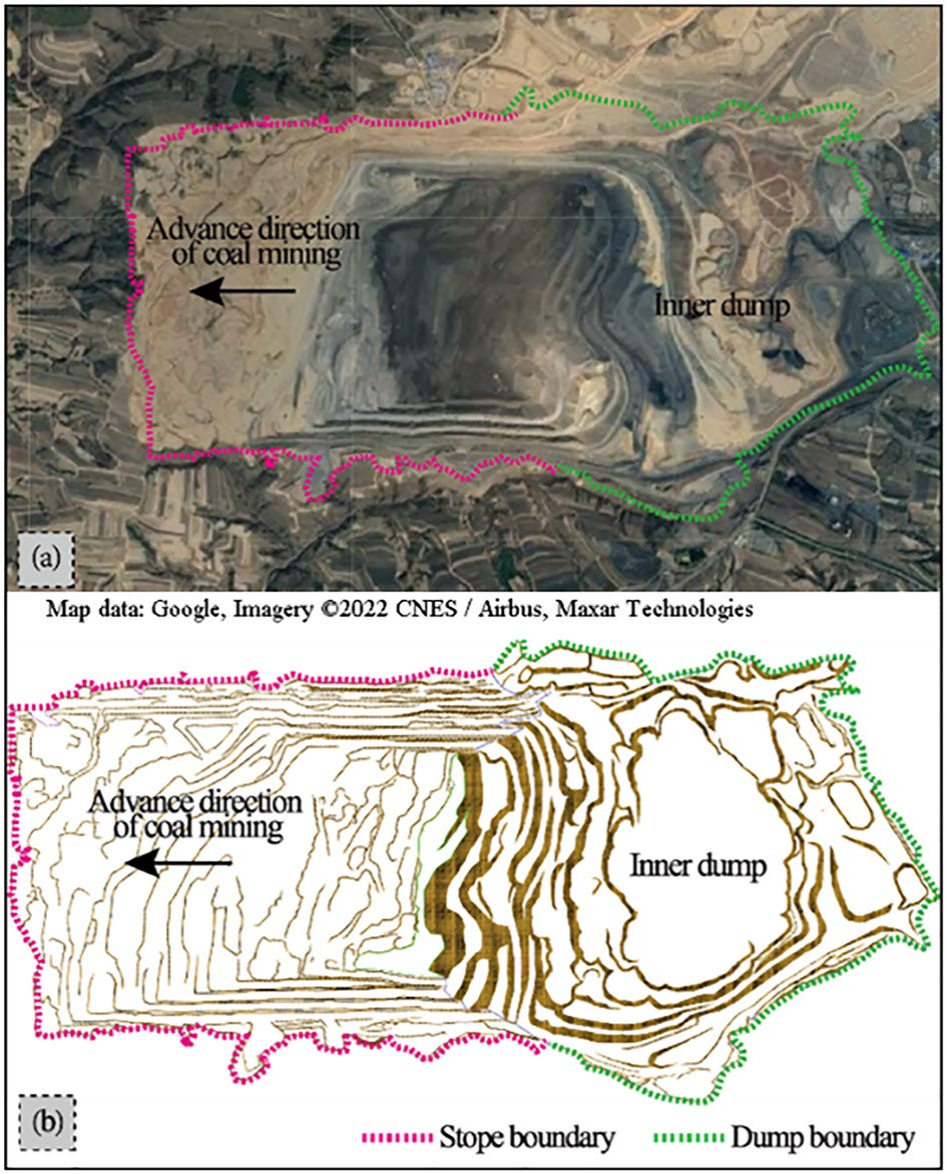
Location of the study area and Geomorphology of the open pit mine: (**a**) Aerial view of the Pingshuo East-Open pit mine (Satellite image source from Google Earth); (**b**) Open pit mine production status model diagram (3DMine Mining Engineering Software Version 11.6). The range circled in red is the stope boundary, and the range circled in green is the dump boundary. Arrows indicate the advance direction of coal mining.

Pingshuo East Open-pit Coal Mine is located at the northern end of Ningwu Coalfield, which is under the jurisdiction of Pinglu District, Shuozhou City. It is about 10 km northeast of Pinglu District, with a surface boundary area of 48.73km^2^ (as shown in Fig. 1) and a geological reserve of 1849 Mt It is another large-scale modern open-pit coal mine after Antaibao and Anjialing Open-pit mines in Pingshuo Mining Area. The designed scale is 20.0 Mta, the annual stripping capacity is 110Mm^3^/a, and the mining boundary is 1.99–5.95 km long from east to west and 4.90–10.63 km wide from north to south.The surface area is 45.90 km^2^, the mining depth is 160–270 m, the dip angle of strata is between 2 and 7, generally below 5, and No.4, No.9 and No.11 in the boundary are the main minable coal seams. The bottom width of the first mining area is 1400 m, and the surface width is 2200 m East open-pit mine belongs to the low hills of Shuopingtai in Shanxi Loess Plateau. The whole area is mostly covered by loess. The rock structure of the mining area is roughly divided into:(1) The flood-flushing base is composed of limestone gravel, pebbles, silty sand, medium and coarse sandstone cuttings and proluvial, with a thickness of 0–40 m and an average thickness of 10 m; ② The sub-clay layer is composed of red-yellow sub-clay and paleosol layer, containing calcareous nodules and gravel at the bottom, with a layer thickness of 3.10–47.40 m and an average thickness of 39.64 m;③ The sandy mudstone layer is mainly composed of yellow-green, grayish yellow, gray powder, fine-grained sandstone and medium-grained sandstone, gray, yellowish-green sandy mudstone, dark gray, grayish black sandstone, sandy mudstone and mudstone, with a thickness of about 101.36 m Most of the rocks are semi-hard rocks with a certain number of weak and hard layers.

The main minable coal seams in the east open-pit mine are thick, the coal quality in the mining area is high, and the coal seams are shallowly buried, which is suitable for open-pit mining. However, due to the large-scale mining of small coal mines, a large number of mined-out areas remain in the open-pit boundary of current production, and the mined-out areas are affected by ground pressure, rock weathering and blasting vibration for a long time, and a large number of reserved pillars have been destroyed. The size, height and roof stability of goaf have changed.

### Hydrogeological conditions

Although the water content in some areas of this mining area is relatively strong, due to the simple geological structure and more finely divided rocks, the groundwater in bedrock is basically absent, that is, undeveloped, the water conductivity, water seepage and water-rich properties of rocks are weak, and the hydrogeological conditions are simple. The main research object is the division and characteristics of the direct aquifer at the top of 4# coal seam, which is described as follows:

The water-bearing section of Quaternary loose layer is mainly composed of aeolian sand and gravel, with a thickness of 0–40.58 m and a general thickness of 16.7 m, and the distribution thickness of the whole area varies greatly. Generally, the spring water flow is less than 0.79L/S in normal season.

### Formation and development of mined-out areas in mining areas

**Figure 2:**
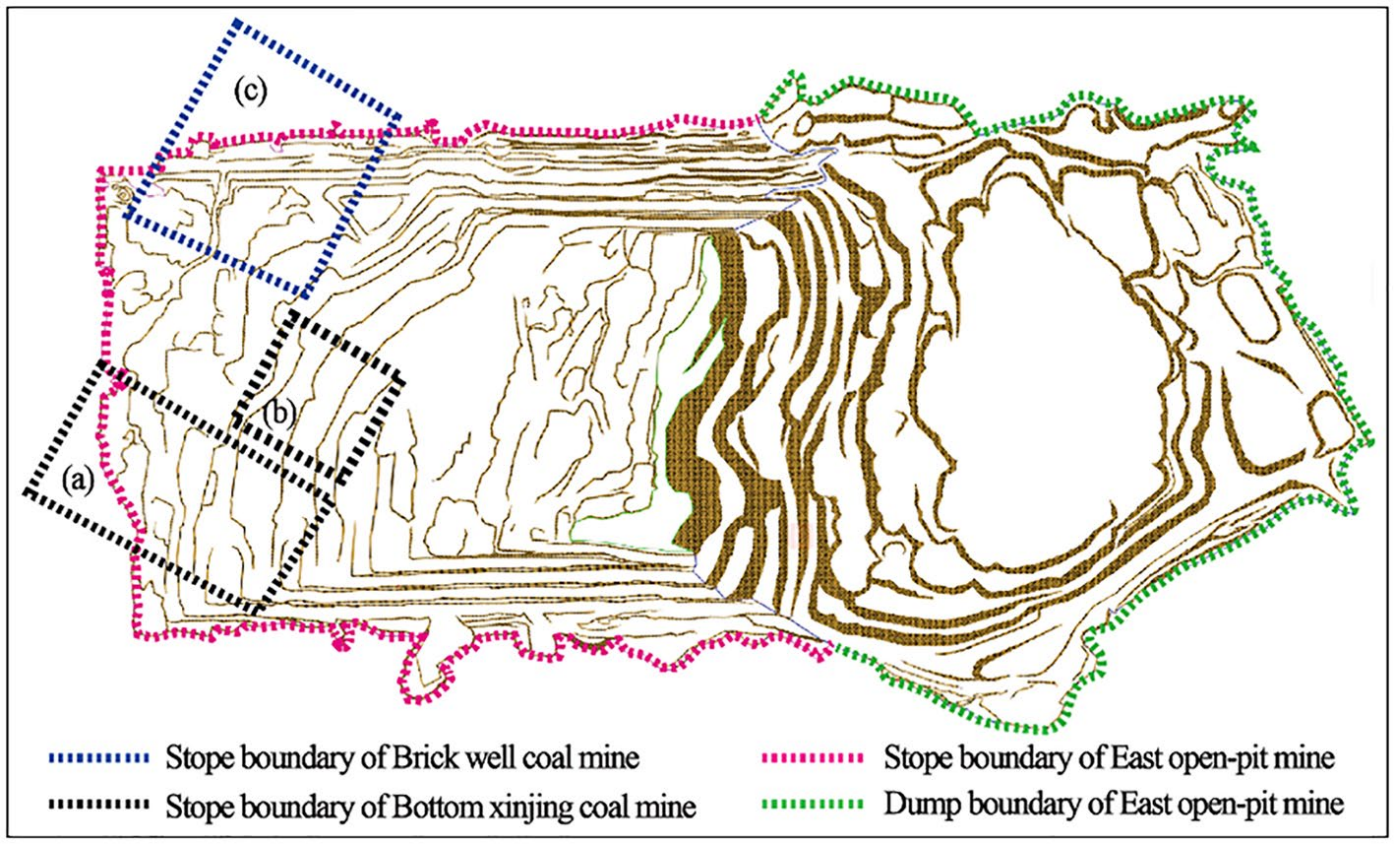
Distribution map of mined-out area of small kiln in the first mining area of East open-pit Mine (3DMine Mining Engineering Software Version 11.6): (**a**,**b**) Stope boundary of Bottom xinjing coal mine; (**c**) Stope boundary of Brick well coal mine.

The mined-out area of the East Open-pit Mine is mainly formed by the mining of mines and small coal mines. At present, there are mainly ditch bottom xinjing coal mine and brick well coal mine in the mining scope of East Open-pit Mine as shown in Fig. 2a–c.

The mined-out area of Dixinjing Coal Mine is about 1,089,995 m^2^. No.4, No.9 and No.11 coal seams are mined in batches, and the approved production capacity is 80,000 tons/year. At present, some information about the mining situation of No.4 coal seam and No.9 coal seam has been collected. The mined-out area of Zhuanjing Coal Mine is about 145,392 m^2^. No.4 coal seam was mined, and the approved production capacity was 150,000 tons/year. At present, some information about the mining situation of No.4 coal seam in 2008 has been collected.

The small kiln mining in the first mining area of East Open-pit Mine is roughly divided into three stages, with the mining scale gradually increasing and the transportation equipment gradually becoming advanced.

In the first stage (1996–1999), room and pillar stopping was adopted, and the mining area was deployed once and twice. The length of the stopping face was 50 m, the cut-off interval was 15 m, the coal pillar was 7 m in the stopping face, the mining width was 8 m, the stopping height was 9–10 m, and the coal pillar was 5 m in the gateway lane and the return air lane. The mining sequence is open-cut, side expanding, roof capping, coal raking, and trucking (manual coal loading and mule cart transportation).

In the second stage (2000–2004), room and pillar stopping was adopted, and the mining area was deployed once and twice. The length of the working face was 50 m, the cut-off interval was 16–18 m, the coal pillar left in the working face was 7 m, the mining width was 9–10 m, and the mining height was 9–10 m. Before and after the gateway lane and return air lane, each coal pillar is 5 m. The haulage gateway is a series of cars, and some are tricycles.

In the third stage (2004–2008), room and pillar stopping was adopted, and the mining area was deployed once and twice. The length of the stopping face was 50 m, the cut-off interval was 20–25 m, the coal pillar was 7 m in the stopping face, the mining width was 14–19 m, the stopping height was 11–12 m, and the coal pillar was 5 m in the gateway lane and the return air lane. The mining sequence is: open-cut, side expanding, coping, coal stripping and belt transportation. Transport gateway is 40 type scraper conveyor and 80 type belt conveyor. The main transportation lane is a 100-type belt conveyor.

## Methodology

### The principle of making empty area detection scheme

The experiment was mainly carried out in the East Open-pit Mine, and the relationship between the spatial position of stope slope and goaf is complicated, which makes it difficult to accurately determine the position and shape of goaf, and it is also a great challenge to determine a suitable goaf detection scheme. In view of the above reasons and characteristics, the following principles should be followed when formulating the goaf detection scheme:

(1) Safe and convenient. Because of the age of goaf formation and the complex geometry of goaf, the stability of surrounding rock in goaf is getting worse and worse, so surveyors can't enter the goaf to detect the goaf, so the ground detection method should be preferred.

(2) The instrument has good adaptability. The surface topography above the mined-out area is generally complex, which can not meet the layout requirements of some detection instruments. When selecting the detection method of the goaf, we should fully grasp the surface topography above the goaf, carry out the feasibility test of the detection instruments and methods, and then select the appropriate detection methods and instruments.

(3) Low risk and low cost. On the premise of ensuring the detection accuracy, the selection of detection method and the formulation of detection scheme should minimize the disturbance and influence on the overlying rock mass of goaf, and reduce the detection cost by reducing part of the drilling workload.

(4) High accuracy. The physical properties of the strata where the mined-out area is located are different. Therefore, reasonable exploration methods should be targeted to obtain reliable detection accuracy.

(5) The scheme is reasonable. When making the goaf detection scheme, we should fully consider the complexity of the surface topography above the goaf, give full play to the joint advantages of various detection methods, and give the optimal survey line layout and the row spacing of drilling holes, so that the detection results obtained by various detection methods can be mutually verified. Form an ideal balance point between detection accuracy and detection cost.

Table 1 shows the advantages, disadvantages and application conditions of various geophysical methods. According to different engineering geological conditions and research contents, one or more geophysical methods can be selected according to Table 1. In view of the complexity of the old goaf, it is difficult for a single geophysical method to accurately detect the complex goaf, but multiple geophysical methods should be used for joint detection.The best balance between detection accuracy and detection cost can be achieved.

**Table 1:** Several common goaf detection methods divided according to working range, type, method, detection depth and their characteristics. a joint detection method suitable for complex goaf is selected, that is, Transient electro-magnetic method, Ground penetrat-ing radar and High-density elec-trical method.

Working range	Type	Method	Detection depth (m)	Applicable conditions and advantages and disadvantages
Above ground	Electrical method	Ground penetrating radar	0–20	It is suitable for the surface with relatively flat surface and no low resistance layer (water, clay) shielding, and has a certain degree of feedback on the scale, shape and filling of cavity development, with high resolution. It is greatly affected by surface physical conditions and topography
		Transient electromagnetic method	10–1000	From the field and engineering effect, its detection depth is high, for low resistance geological body, can get a good detection effect, and can achieve outstanding feedback to groundwater, in addition to the surface terrain conditions of low requirements; There is a blind area in shallow detection, which is prone to electromagnetic interference and has low resolution
		EH4	70–1000	From the field and engineering effect, its detection depth is high, easy to overcome the irregular situation of the ground; The blind area is relatively large and susceptible to electromagnetic interference
		High-density electrical method	0–50	I The best overburden layer is thin, which requires high topographic conditions on the surface, and the length of the arrangement limits the depth of detection
Under the ground	Earthquake wave	Shallow seismic wave method	0–50	The goaf deep in bedrock cannot be well detected, and it has high requirements on the surface topographic conditions. When the surface is undulating, it is prone to false anomalies, and the work efficiency is low
	CT imaging between boreholes	Drilling electromagnetic wave CT	10–50	It can well detect the goaf between boreholes and has a high horizontal resolution. Increasing the depth of boreholes can improve the depth of detection. The work efficiency is low, have certain limitation
		Earthquake wave CT	10–50	It can distinguish the properties of filling materials in goaf with high resolution and large penetration distance. Water is needed to be used as coupling agent
		Resistivity CT method	0–100	Electrode power detection needs water as a coupling agent, drilling can not store water, can not be detected
	Single aperture optical imaging	Borehole television imaging	Wall of hole	It can intuitively observe the size, geometry and filling properties of goaf with clear imaging

### Joint geophysical prospecting technology

In view of the characteristics of many underground small coal mines and many mined-out areas in the East Open-pit Mine, the underground joint detection technology was adopted on the spot, that is, TEM, GPR and HRM technology and geological drilling were used to fully explore the mining boundary and land requisition range. The distribution characteristics, buried depth and other conditions of mined-out areas within the research scope of East Open-pit Mine are found out by the joint geophysical prospecting technology,In order to accurately describe the characteristics of the cavity in the goaf, the information of the cavity in the goaf can be obtained by using 3D laser scanning technology. The goaf is drilled by geological drilling technology. According to the quality of the drilled hole, the 3D laser scanning instrument is placed inside the goaf for scanning, and the information inside the cavity in the goaf is processed. With the help of geological software,Drawing the 3D geometric map of the goaf in the computer can further master the key parameters and point cloud data of the goaf, such as geometric shape, projected area, 3D volume, goaf height, overlying rock thickness, etc., and construct the 3D geometric model and 3D geological model of the goaf, which can provide the basis for the subsequent goaf stability analysis and goaf governance.

There are a large number of underground mined-out areas in East Open-pit Mine. Only one exploration method can not accurately obtain the information of mined-out areas. For example, the seismic reflection wave method (TEM) uses artificial blasting, that is, artificial wave. As the production operation goes on, heavy equipment will cause clutter, and this information will also be received by instruments, and the results will definitely have a certain gap with the actual situation. Therefore, using joint detection technology, the suspected mined-out area is detected again. According to the detection results of different instruments, repeated verification and repeated comparison are carried out to determine the existence of mined-out area. In this study, combined with the actual engineering situation, the following detection methods are adopted:

### Seismic reflection wave method

**Figure 3:**
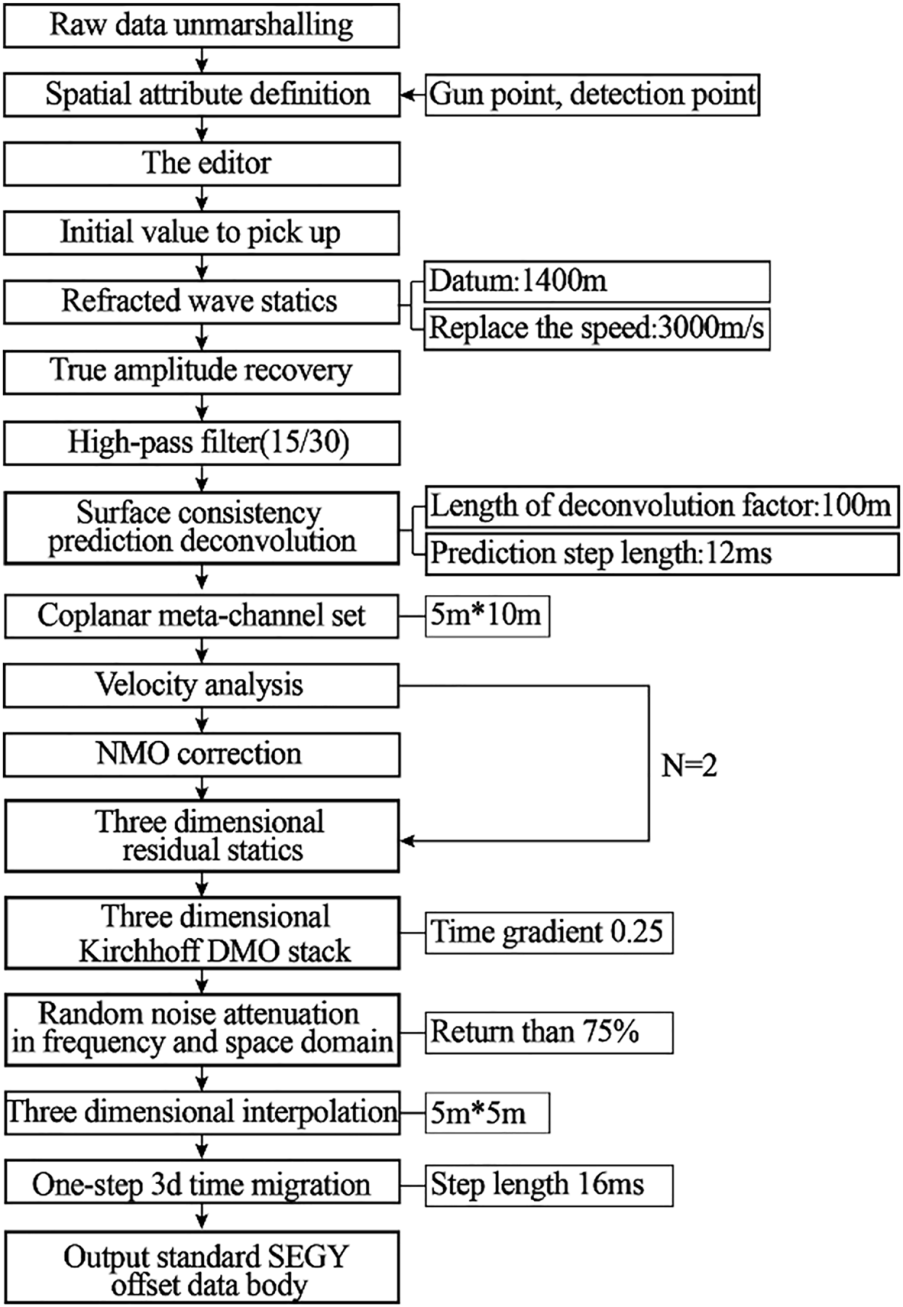
Processing flow chart. process of key parameters processing and analysis.

Three-dimensional seismic exploration is a data acquisition and processing system that fully samples geological bodies. Using scalar wave equation to image the acoustic reflectivity of rock mass can accurately reflect the structure and lithology of rock mass. The basic principle of seismic exploration methods is to generate elastic waves by source control to obtain images of underground geological bodies. Seismic waves are strain energy pulses propagating in solids and liquids. Earthquake energy, whether on the earth's surface or in shallow borehole, will produce the following wave patterns: (1) body wave, in which energy is transported in all directions. (2) Surface wave, where energy propagates along or near the surface. Through the processing and analysis of seismic wave, draw it into a section,Then interpret the geological structure and other information, and obtain the physical parameters of underground geological bodies. Combined with the specific situation of seismic exploration, extensive tests and special studies were carried out, and the process of key parameters processing and analysis was given, as shown in Fig. 3.

### Transient electromagnetic method

**Figure 4:**
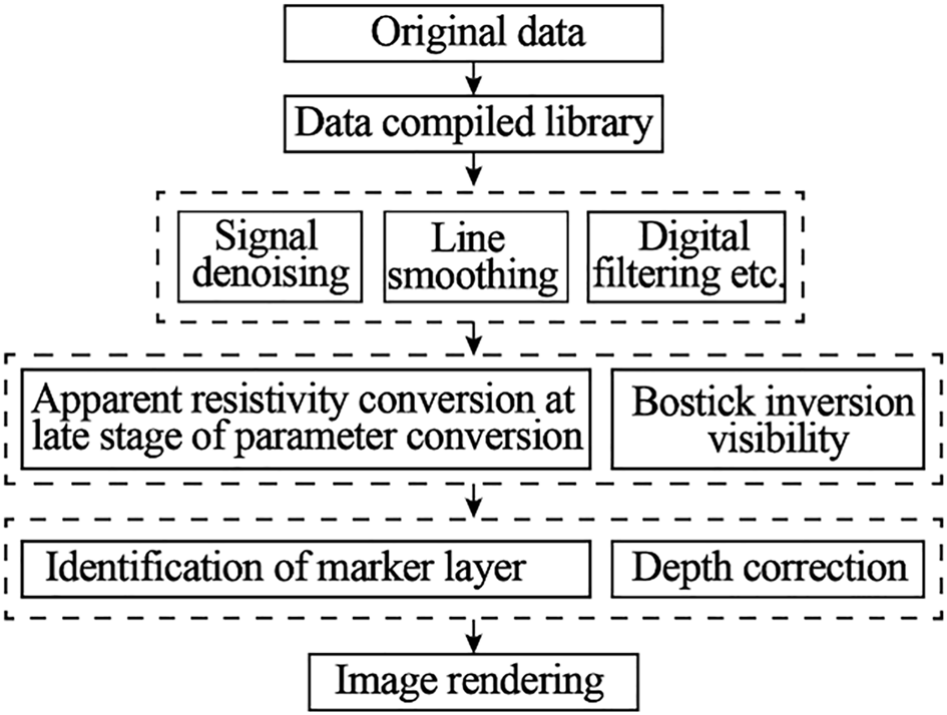
Transient electromagnetic data processing flow.

Transient electromagnetic method is also called time domain electromagnetic method, or TEM for short. It is an electromagnetic method that uses artificial signals generated by specific transmitters to measure in time domain. The current in the transmitter circuit was suddenly cut off. According to the law of electromagnetic induction, the collapse of electromagnetic field induced eddy current in the conductive underground, which further produced secondary magnetic field.Then, the coil or electrode is used to observe the spatial distribution characteristics, intensity and time characteristics of the secondary eddy current field. The working principle is shown in Fig. 4.

Transient electromagnetic instrument is used to record, process and analyze normalized induced secondary field, emission current, sampling time, etc., so as to obtain relevant information that can reflect underground geological bodies.

### High damping resistivity method

Electrical exploration is a valuable exploration method to explore various types of deposits, find out geological structures and analyze related geological problems by observing, analyzing and studying the "time–space" distribution and evolution characteristics of electric field, chemical field or electromagnetic field according to the differences of electromagnetic properties (such as dielectric, magnetic conductivity and conductivity) and electrochemical characteristics of different rock bodies or ore bodies.

The high-density resistivity method is still an exploration method based on the electrical difference of rock mass, and its principle is exactly the same as that of the conventional resistivity method, which is a branch of the conventional resistivity method. Similarly, analyzing and studying the "time–space" distribution and evolution characteristics of underground electric field can be used to solve the problems of mineral resources, engineering geology and environment. As an array exploration method,When detecting in the field, about 100 electrodes are installed on the measuring points, and the detection data can be efficiently collected by electric measuring instrument and change-over switch. Then, through comparison, analysis and interpretation, different results maps are finally formed.

### Geological drilling exploration

Geological drilling exploration can establish the most direct connection with the mined-out area. According to the results of geophysical exploration, determine the information of the suspected mined-out area, and drill holes above the mined-out area by using geological drilling exploration technology, so as to verify the existing information of the mined-out area and finally define the results of geophysical exploration. Besides determining the information of the mined-out area,Geological exploration can also provide necessary conditions for 3D laser scanning.

## Results

### Analysis of results of seismic reflection wave method

**Figure 5:**
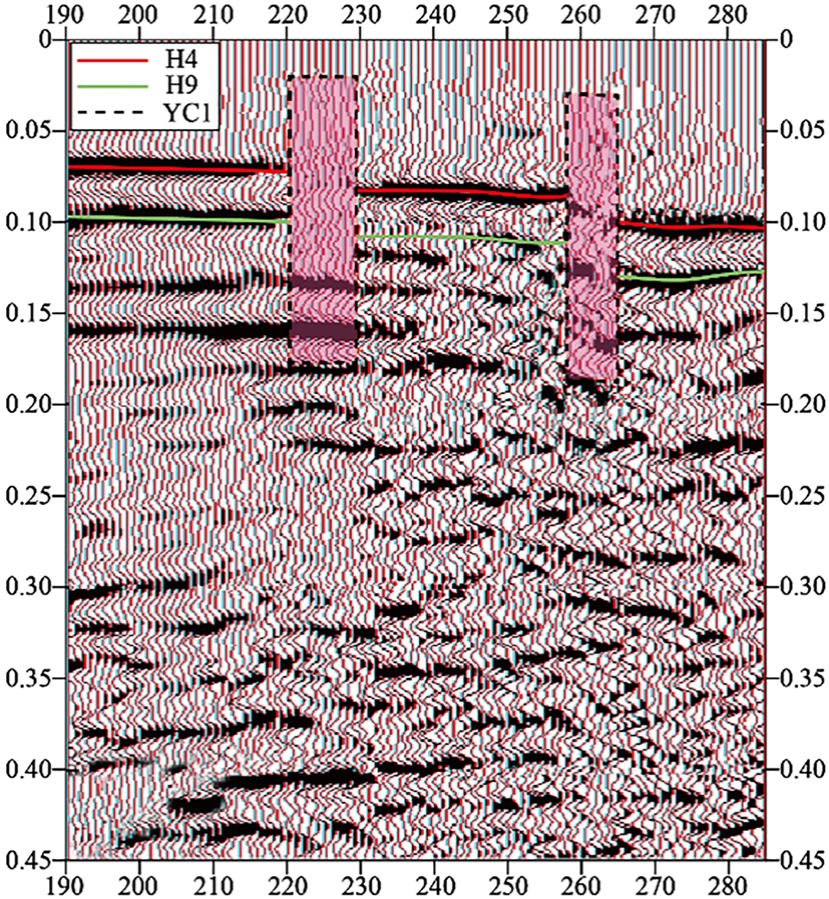
Typical time section of mined-out area in small coal mine.

**Figure 6:**
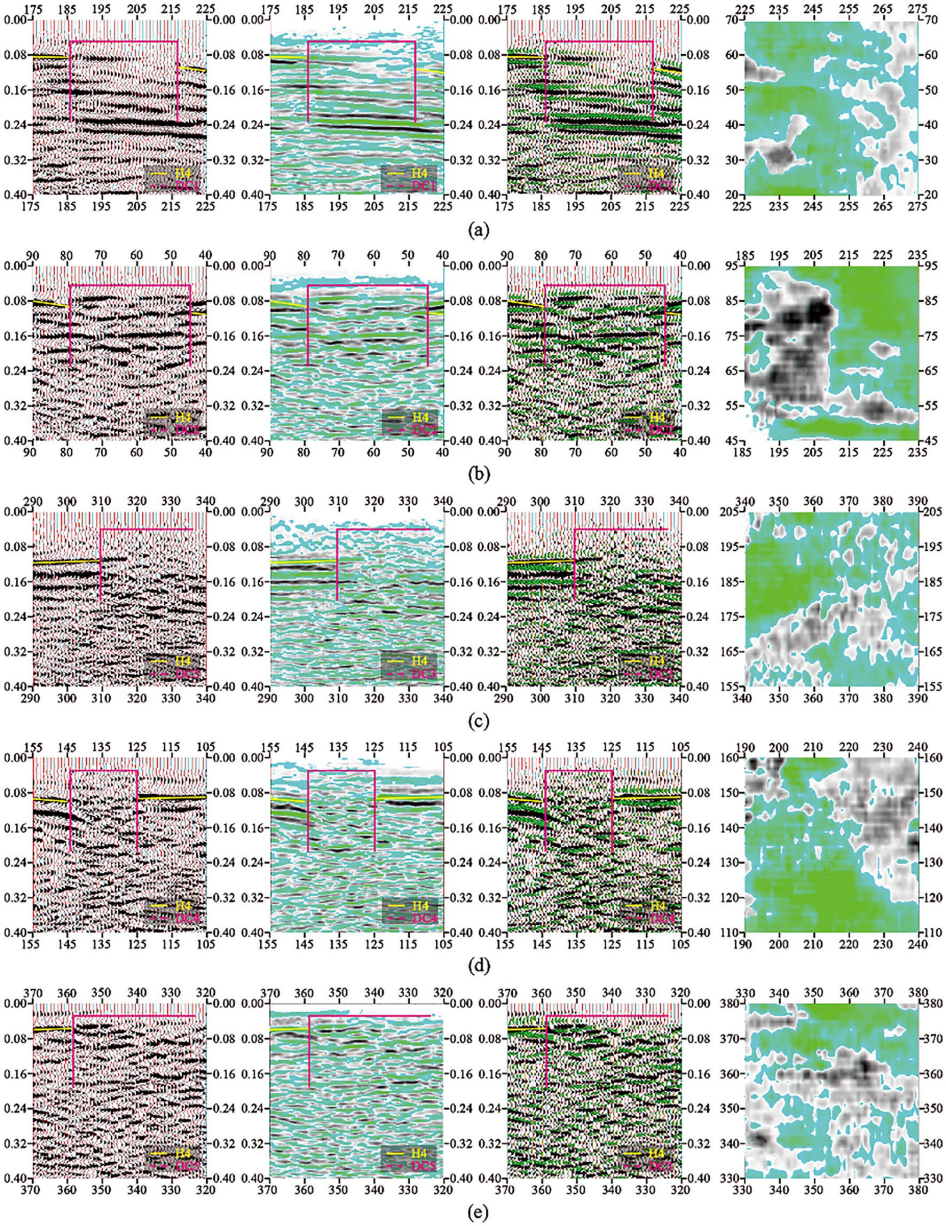

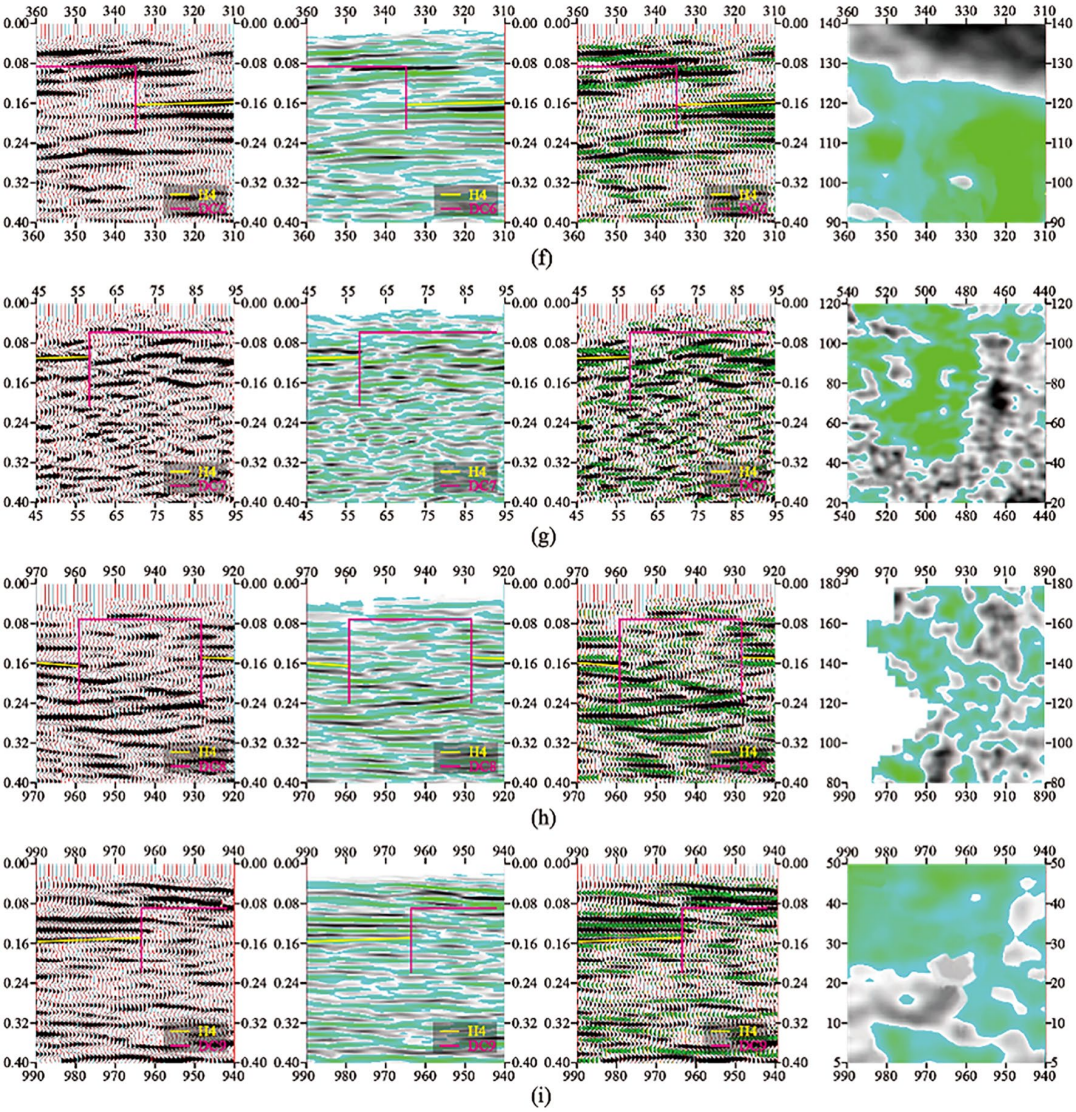
Schematic diagram of interpretation of inferred goaf (3D seismic exploration analysis and detection equipment): (**a**) DCI comprehensive interpretation schematic diagram; (**b**) DC2 comprehensive interpretation schematic diagram; (**c**) DC3 comprehensive interpretation schematic diagram; (**d**) DC4 comprehensive interpretation schematic diagram; (**e**) DC5 comprehensive interpretation schematic diagram; (**f**) DC6 comprehensive interpretation schematic diagram; (**g**) DC7 comprehensive interpretation schematic diagram; (**h**) DC8 comprehensive interpretation schematic diagram; (**i**) DC9 comprehensive interpretation schematic diagram. inferred mined-out areas are clearly reflected in abnormal seismic time profiles.

**Figure 7:**
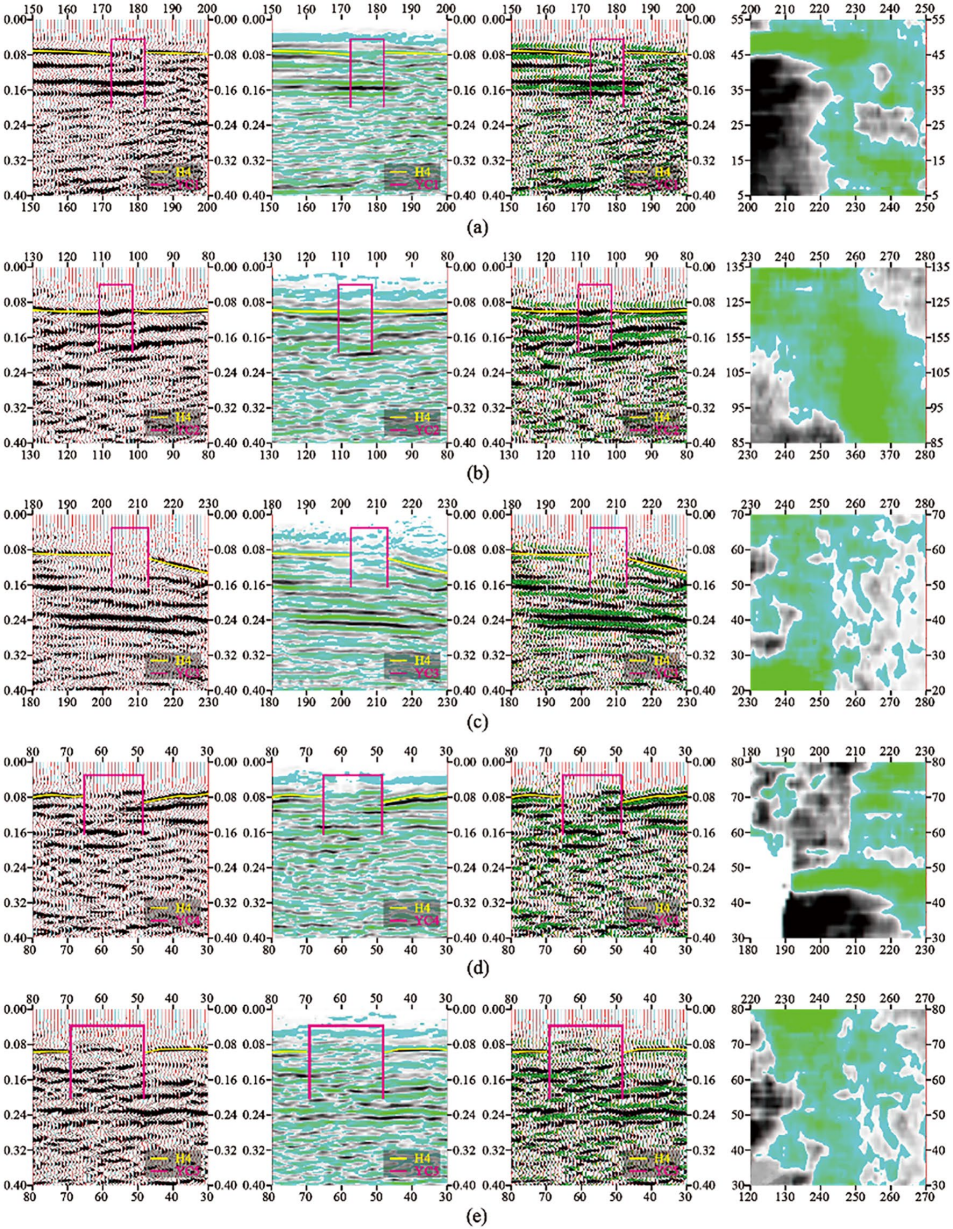

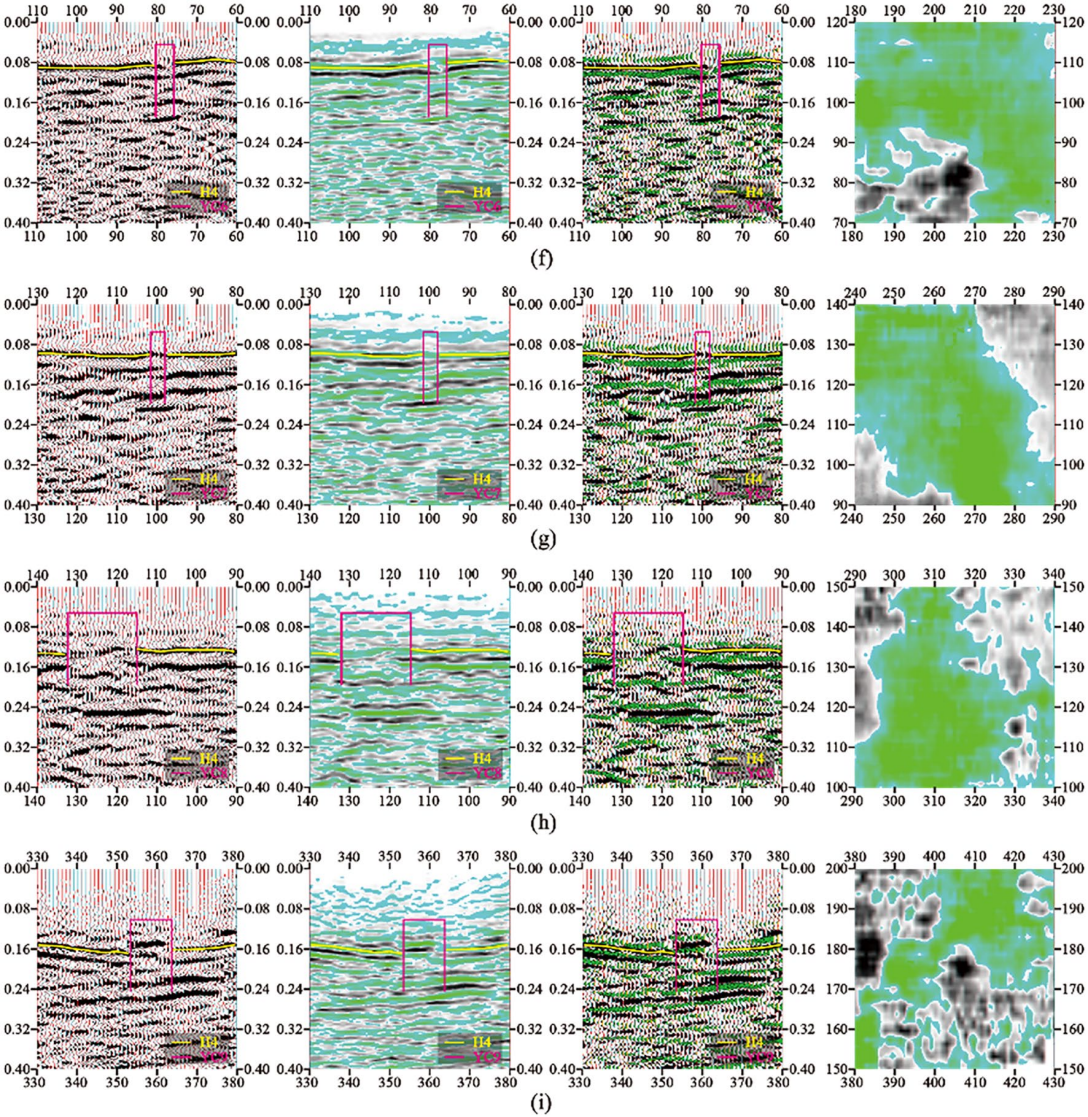
Schematic diagram of interpretation of suspected goaf (3D seismic exploration analysis and detection equipment): (**a**) YC1 comprehensive interpretation schematic diagram; (**b**) YC2 comprehensive interpretation schematic diagram; (**c**) YC3 comprehensive interpretation schematic diagram; (**d**) YC4 comprehensive interpretation schematic diagram; (**e**) YC5 comprehensive interpretation schematic diagram; (**f**) YC6 comprehensive interpretation schematic diagram; (**g**) YC7 comprehensive interpretation schematic diagram; (**h**) YC8 comprehensive interpretation schematic diagram; (**i**) YC9 comprehensive interpretation schematic diagram. compared with inferred mined-out areas, suspected mined-out areas are not clearly reflected in abnormal seismic time profiles.

The total working area of seismic exploration in this study is 3.11 km^2^, which can be divided into the following three areas: the self-operated mining and stripping area of the first mining area of East Open-pit Mine (the first task, with an area of 0.94 km^2^), the outsourcing stripping area to the west of the self-operated mining and stripping area of the first mining area (the second task, with an area of 0.51 km^2^) and the coal haulage roadway area to the west of the central part of the southern end of the mine (the first task, with an area of 0.5 Organize and process the data obtained from this 3D seismic exploration. The analysis of the detection data shows that the signal-to-noise ratio is relatively general, the first break wave in the collected data is relatively clear, and some random interference may be generated by large mining equipment and vehicles, with acoustic waves and surface waves as the main interference waves. The quality of the collected data is comprehensively assessed as general.The interpretation of geological structure in this exploration is mainly carried out from three aspects: the inference of goaf, the inference of abnormal area of collapse column and the detection results of seismic evaluation. The comprehensive evaluation is as follows:

The typical time profile of the selected goaf section of small coal mines is shown in Fig. 5. As shown in Fig. 6, in the research area with a total area of 3.11 km^2^, 9 inferred mined-out areas and 9 suspected mined-out areas were found by using 3D seismic exploration method. It is concluded that the characteristics of mined-out area and suspected mined-out area are the same: on the seismic event profile, the frequency of reflected wave becomes lower, The in-phase axis is disordered and obviously trapped on the horizontal isochronous slice. The difference between inferred mined-out areas and suspected mined-out areas lies in that inferred mined-out areas are clearly reflected in abnormal seismic time profiles, while suspected mined-out areas are not clearly reflected in abnormal seismic time profiles. Illustration of typical inferred goaf and suspected goaf, As shown in Figs. 6 and 7.

### Analysis of detection results of transient electromagnetic method

There are 6,900 physical points of transient electromagnetic exploration, with a total area of 2.65 km^2^, which are mainly carried out in two stages: (1) Self-operated mining area by the working party (in the first stage, there are 2,444 measuring points with an area of 0.94 km^2^). (2) The stripping area is outsourced to the west of the self-operated stripping area of the first mining area (the second stage, there are 4,456 measuring points with an area of 1.71 km^2^).

The post-processing process of transient electromagnetic detection includes the following steps: first, preliminary processing of the detected data; Step 2, sift the waveform results to eliminate the influence of useless signals and show the original features of geological signals; Step 3, using special software to transform the detection parameters to obtain the apparent depth, apparent resistivity and other indicators; Step four,Refer to the relevant data of drilling, geology and survey, and correct the elevation and topography; The fifth step is to draw contour map and section map, which can be used for anomaly reliability evaluation, anomaly division and physical parameter statistics. The above process can be simplified into two major steps: the first step,The intermediate results for qualitative analysis (TEM curve type plan) are formed by processing the data collected in the field; The second step is to analyze and interpret the intermediate results to form results maps (apparent resistivity plan and apparent resistivity section).

**Figure 8:**
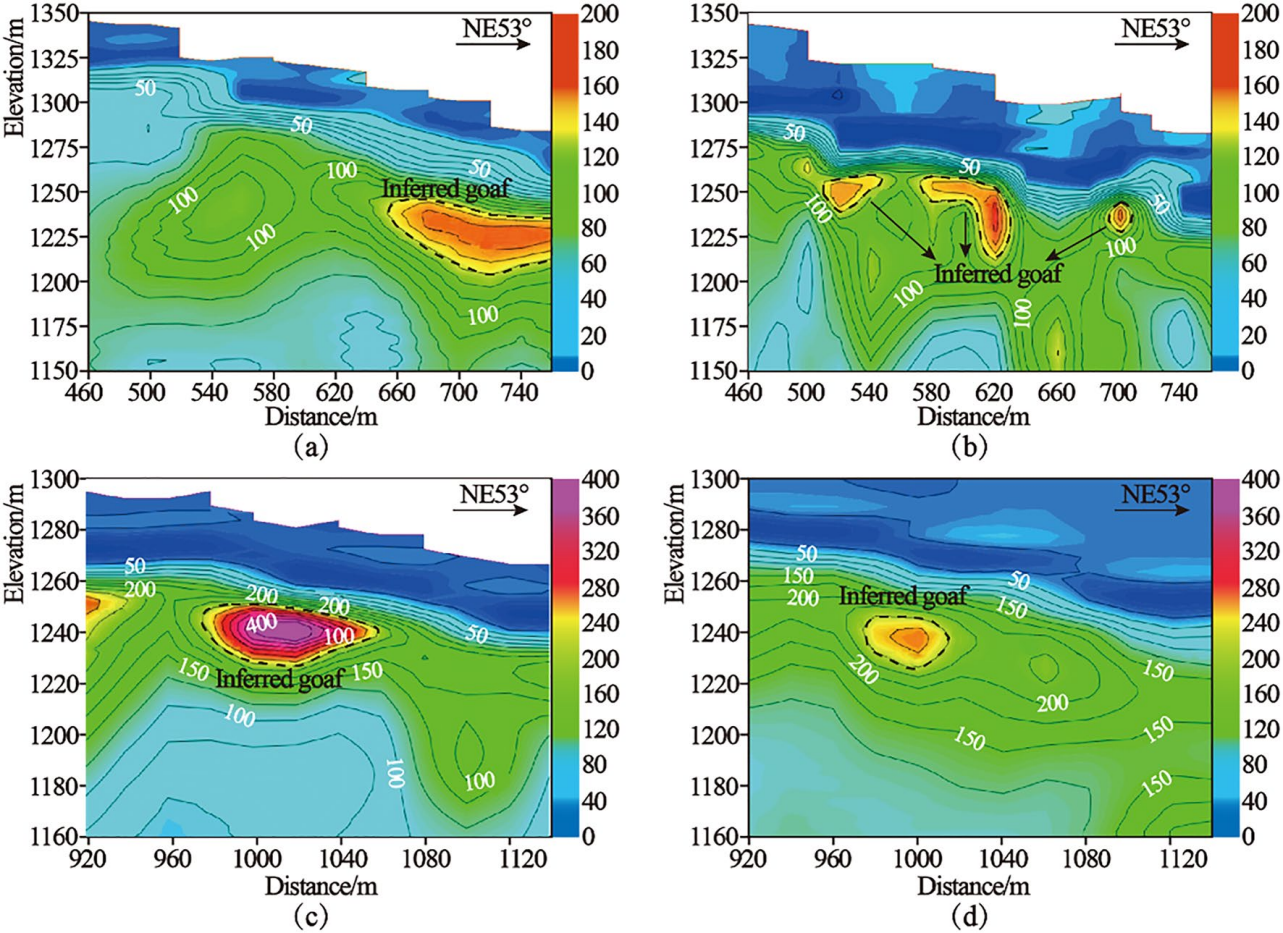
Composite sections of typical apparent resistivity: (**a**) Integrated section of apparent resistivity of S23 line; (**b**) Integrated section of apparent resistivity of S25 line; (**c**) Integrated section of apparent resistivity of S51 line; (**d**) Integrated section of apparent resistivity of S54 line.

Because the electrical reflection of mined-out area is compared, when inferring the abnormality of geological body, we should not only consider the continuity of resistivity change, but also consider the particularity of geological, drilling, topography and other conditions, compare with the field geological survey and field exploration records, coordinate the evolution law of plan and cross-sectional drawings, and complete comprehensive and systematic induction and summary.Through the above steps, the detection area is determined, as shown in Table 2 and the detection result is shown in Fig. 8. 223 specific areas have been delineated, and some specific areas have been actually verified with the continuous production.

**Table 2:** The inflection point coordinates and area of Transient electromagnetic exploration.

Division of transient electromagnetic exploration stages	Coordinates of inflection point in exploration area			Area(km^2^)
	Serial number	X	Y	
Phase one phase one(A-region )	1	495,161.28	4,379,866.29	0.386
	2	495,217.46	4,379,908.04	
	3	495,265.18	4,379,843.82	
	4	495,393.6	4,379,939.25	
	5	495,584.46	4,379,682.4	
	6	495,656.7	4,379,736.08	
	7	495,513.56	4,379,928.72	
	8	495,569.74	4,379,970.47	
	9	495,897.78	4,379,529.01	
	10	495,833.57	4,379,481.29	
	11	496,023.55	4,379,225.51	
	12	495,960.17	4,379,176.79	
Phase one phase one(B-region )	1	495,657.32	4,380,079.07	0.089
	2	495,689.59	4,380,102.87	
	3	495,725.18	4,380,054.7	
	4	495,821.55	4,380,126.37	
	5	496,024.27	4,379,853.39	
	6	495,976.07	4,379,817.73	
	7	496,011.88	4,379,769.54	
	8	495,963.66	4,379,733.72	
	9	495,999.45	4,379,685.62	
	10	495,919.66	4,379,625.17	
	11	495,680.93	4,379,946.18	
	12	495,728.85	4,379,982.8	
Phase two	1	495,357.03	4,378,236.4	1.53
	2	495,999.16	4,378,713.55	
	3	495,295.36	4,379,660.69	
	4	495,215.1	4,379,601.04	
	5	494,714.09	4,380,275.28	
	6	494,521.45	4,380,132.13	
	7	494,449.88	4,380,228.45	
	8	494,176.98	4,380,025.66	
	9	494,463.26	4,379,640.39	
	10	494,366.95	4,379,568.82	
Total area				2.005

Divided into three stages, that is, Phase one phase one(A-region ) , Phase one phase one(B-region ) , Phase two. The region of each stage consists of inflection point coordinates.

### *High *damping* resistivity method*

**Figure 9:**
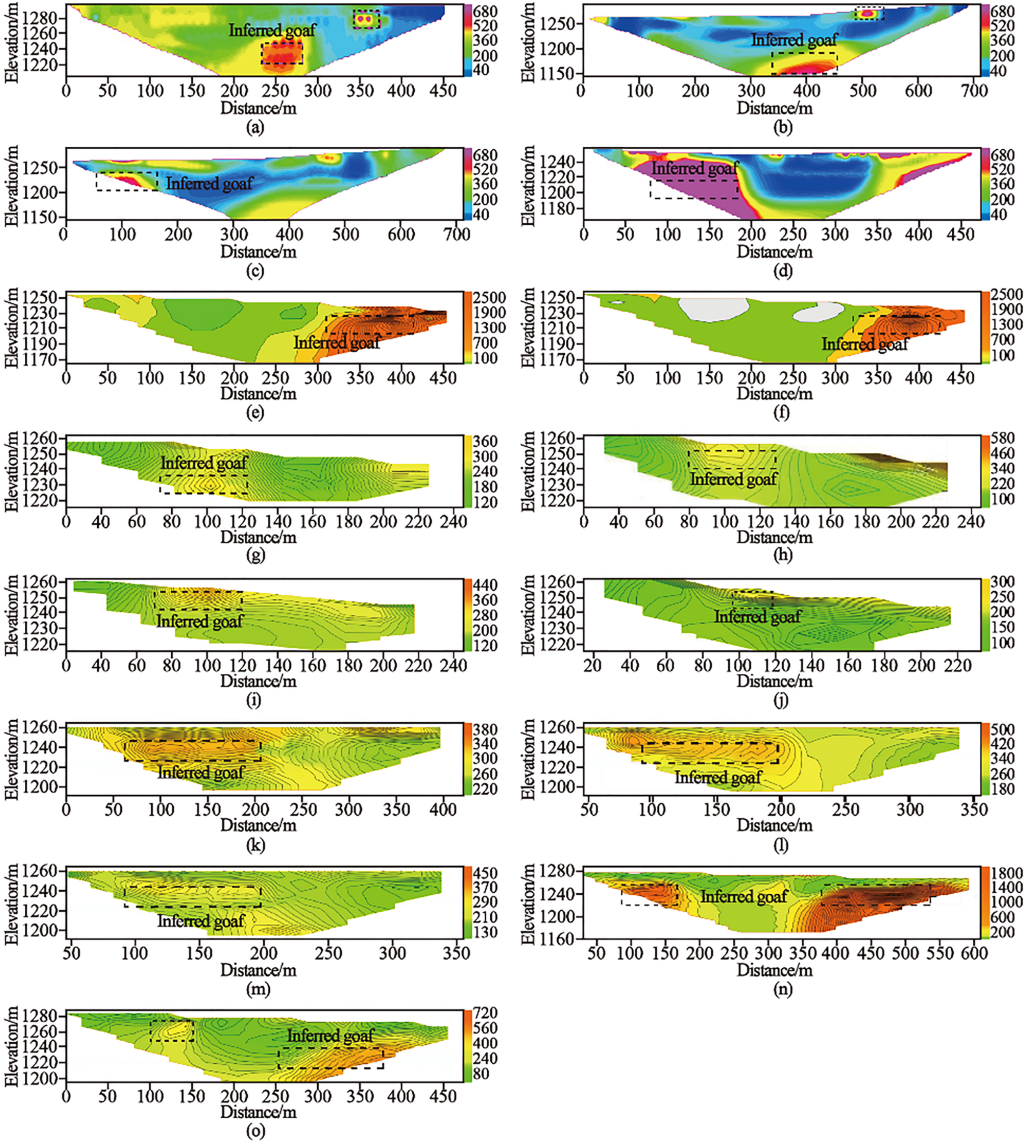
Typical apparent resistivity profiles of high density electrical exploration: (**a**) Apparent resistivity profile of line G0; (**b**) Apparent resistivity profile of line G3; (**c**) Apparent resistivity profile of line G4; (**d**) Apparent resistivity profile of line G5; (**e**) Apparent resistivity profile of line G11; (f) Apparent resistivity profile of line G12; (**g**) Apparent resistivity profile of line G13; (**h**) Apparent resistivity profile of line G14; (**i**) Apparent resistivity profile of line G15; (**j**) Apparent resistivity profile of line G16; (**k**) Apparent resistivity profile of line G17; (**l**) Apparent resistivity profile of line G18; (**m**) Apparent resistivity profile of line G19; (**n**) Apparent resistivity profile of line G20; (**o**) Apparent resistivity profile of line G21. The outline in the profiles show the location of the inferred goaf.

Due to the limitations of the actual conditions such as site topography, slope contour and flat plate length, this exploration work is only carried out in local flat plate, with 630 physical points completed and the exploration area is about 0.3 km^2^. See Table 3. The detection results are shown in Fig. 9. The results of high-density electrical prospecting, 3D seismic prospecting and transient electromagnetic prospecting can well verify and complement each other.

**Table 3:** HRM exploration scope and area.

High density electrical exploration area	Coordinates of inflection point in exploration area			Area (km^2^)
	Serial number	X	U	
High density electrical survey	1	495,708.79	4,380,263.06	0.3
	2	495,493.2	4,380,269.39	
	3	495,465.07	4,380,235.03	
	4	495,481.32	4,380,143.84	
	5	496,039.3	4,379,144.45	
	6	496,209.29	4,379,352.36	
	7	495,872.61	4,380,055.59	
	8	495,829.48	4,380,142.06	

The high density electrical exploration area is composed of inflection point coordinates and the exploration area is about 0.3 km^2^.

### Geological* drilling exploration*

Since the mined-out area was discovered in East Open-pit Mine in 2012, the geological drilling exploration method has been adopted so far. According to the results of underground joint geophysical exploration, 58 drilling holes were arranged in the suspected mined-out area of East Open-pit Coal Mine, and geological drilling exploration was added several times in the later period according to the production needs. Through comprehensive analysis of the production data of small coal mines,The key areas of detection tasks can be obtained, and the utility rate of daily detection drilling is effectively improved.

### Distribution characteristics of hidden goaf groups

Through statistical analysis of goaf groups, it is found that the span of goaf is 15–30 m, the maximum span is about 40 m, the vertical height of goaf is 10–15 m, the maximum vertical height is 25 m, the volume of goaf is 500–3500 m^3^, and the maximum volume of goaf is about 7000 m^3^.

The representative mined-out areas in the mined-out area group are as follows:

(1)1275-122 drilling hole to expose goaf (1275 flat plate).

**Figure 10:**
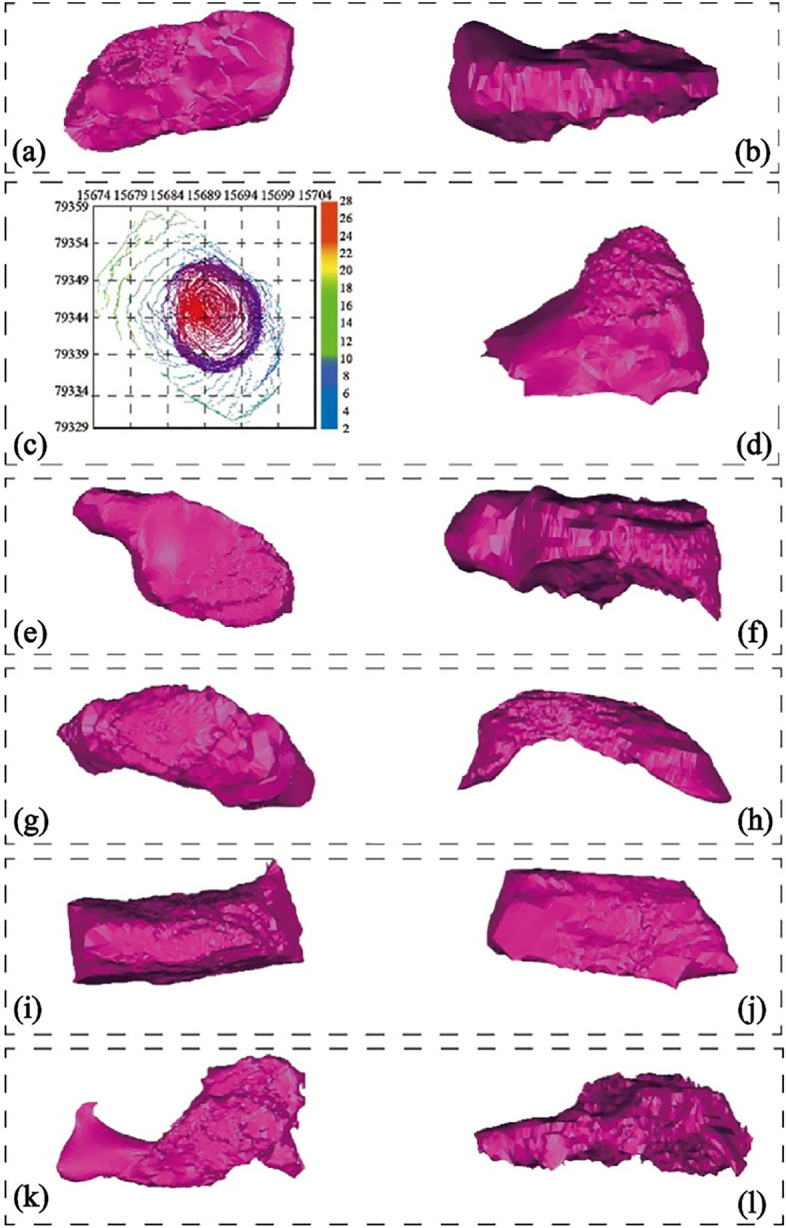
Representative goaf in goaf group: (**a**) Three-dimensional model of short axis of cavity in borehole 1275-122; (**b**) Three-dimensional model of long axis of cavity in borehole 1275-122; (**c**) Three-dimensional model of  major axis of the goaf exposed by the 1320 flat A-59 borehole; (**d**) Three-dimensional model of minor axis of the goaf exposed by the 1320 flat A-59 borehole; (**e**) Three-dimensional model of short axis of borehole 1290-302; (**f**) Three-dimensional model of long axis of borehole 1290-302; (**g**) Three-dimensional model of short axis of goaf exposed by 1290 Pingpan 1290-303 borehole; (**h**) Three-dimensional model of long axis of goaf exposed by 1290 Pingpan 1290-303 borehole; (**i**) Three-dimensional model of major axis of borehole 1275-156 goaf; (**j**) Three-dimensional model of minor axis of borehole 1275-156 goaf; (**k**) Three-dimensional model of short axis of holes 1320-183, 1320-181 and 1320-182; (**l**) Three-dimensional model of long axis of holes 1320-183, 1320-181 and 1320-182.

According to the results of laser 3D scanning, it can be obtained that the volume of the cavity of the go af is about 4850m^3^, the maximum height of the goaf is about 10 m, and the minimum distance from the cavity of the goaf to the ground is 26 m. The mined-out area is oval and distributed from southeast to northwest, in which the short axis is about 20 m and the long axis is about 44 m. As shown in Fig. 10a,b.

(2)A-59 drilling to expose mined-out area (1320 flat plate).

According to the results of laser 3D scanning, it can be obtained that the cavity volume of the goaf is about 3400 m^3^, the minimum thickness of the roof of the goaf from the surface is about 21 m, the major axis of the goaf is about 28.5 m, and the minor axis is about 17.5 m, as shown in Fig. 10c,d.

(3)1290-302 drilling to expose the goaf (1290 flat plate).

From the results of 3D laser scanning, it can be concluded that the cavity volume of the mined-out area is about 4706 m^3^ and the distance from the surface is 33 m. Among them, the mined-out area is distributed from southeast to northwest, the major axis of the mined-out area is about 45 m, the minor axis is about 25 m, the vertical height of the mined-out area ranges from 1246 to 12,585 m, and the maximum vertical height of the roof and floor of the mined-out area is about 10 m, as shown in Fig. 10e,f.

(4)1290-303 drilling to expose the goaf (1290 flat plate).

From the results of 3D laser scanning and geological drilling, it can be analyzed that the cavity volume of this goaf is about 3453 m^3^, the minimum distance from the goaf to the surface is about 34.9 m, and the maximum distance between the roof and floor of the goaf is 10 m. The goaf is distributed in the east–west direction, with the short axis of about 20 m and the long axis of about 48 m, as shown in Fig. 10g,h.

(5)1275-156 drilling hole to expose goaf (1275 flat plate).

The mined-out area is distributed in the northeast direction, with the major axis of about 35 m, the minor axis of about 10 m, the vertical height of the mined-out area ranging from 1,240 to 1,250 m, the vertical heights of the roof and floor of the mined-out area are all about 10 m, and the roof and floor of the mined-out area are nearly horizontal, and the proven volume of the mined-out area is about 1,381 m^3^, as shown in Fig. 10i,j.

(6)1320 flat plate (1320-183 borehole, 1320-181 borehole and 1320-182 borehole).

From the results of 3D laser scanning and geological drilling, it can be analyzed that the cavity of the goaf has a volume of about 6965 m^3^ and a large buried depth, and the maximum distance between the roof and the floor of the goaf is 9 m. The goaf is distributed in the southwest-northeast direction with a short axis of about 19 m and a long axis of about 39 m, as shown in Fig. 10k,l.

**Figure 11:**
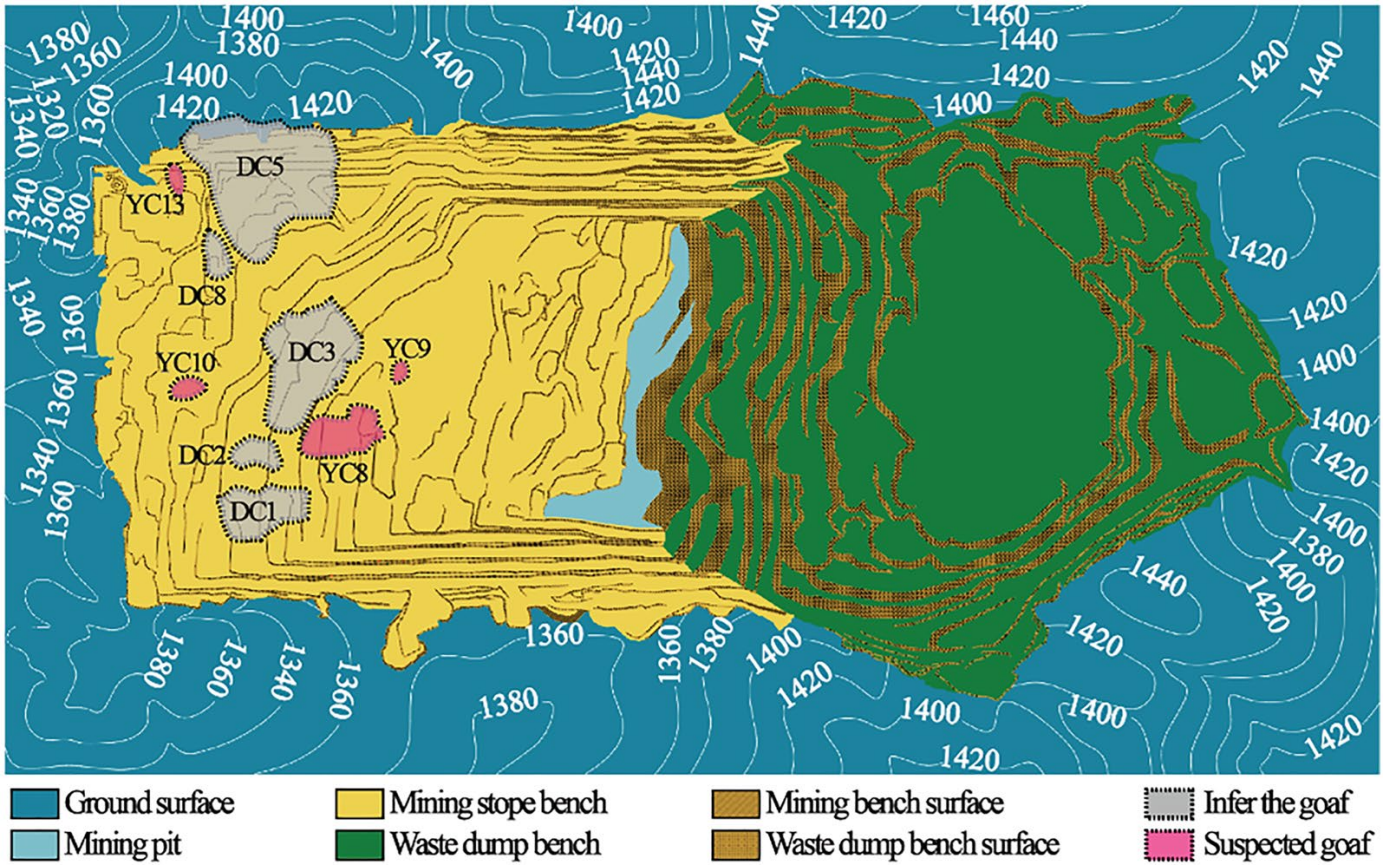
Location of mined-out areas in a mining plan. each area is represented by a different color. The inferred mined-out areas are DC1, DC2, DC3, DC5 and DC8; the suspected mined-out areas are YC8, YC9, YC10, YC13, all of which are displayed in specific colors in the figure.

Based on the above information, the location of the mined-out area is shown in Fig. 11.

### Classification* and formation mechanism analysis of concealed cavity*

The mined-out areas exposed by 3D laser scanning are classified and analyzed. According to the spatial geometry and formation mechanism of mined-out areas, mined-out areas can be classified into three main types. The formation mechanism of goaf and cavity characteristics of goaf can be summarized as follows:

#### Type I

Through statistical analysis, it is found that the discovered mined-out areas are all produced by room and pillar mining in small coal mines. The lithology of overlying rock mass of coal seam is poor. Under the influence of factors such as weathering of rock mass, ground pressure activity and blasting, the damage degree of coal pillar is bound to be serious, the stability of overlying rock mass is constantly decreasing, and the stability of roof above goaf will gradually decrease.Especially, the phenomenon of roof peeling will lead to the upward expansion of goaf and gradually form the geometric shape of "inverted funnel".

The top of the mined-out area is located above the roof of the coal seam. The span of the mined-out area is not large, and there is no obvious characteristic law in its position and height. There is a large free space in the upper part of this kind of goaf, and the ideal effect can be obtained by blasting control measures.

**Figure 12:**
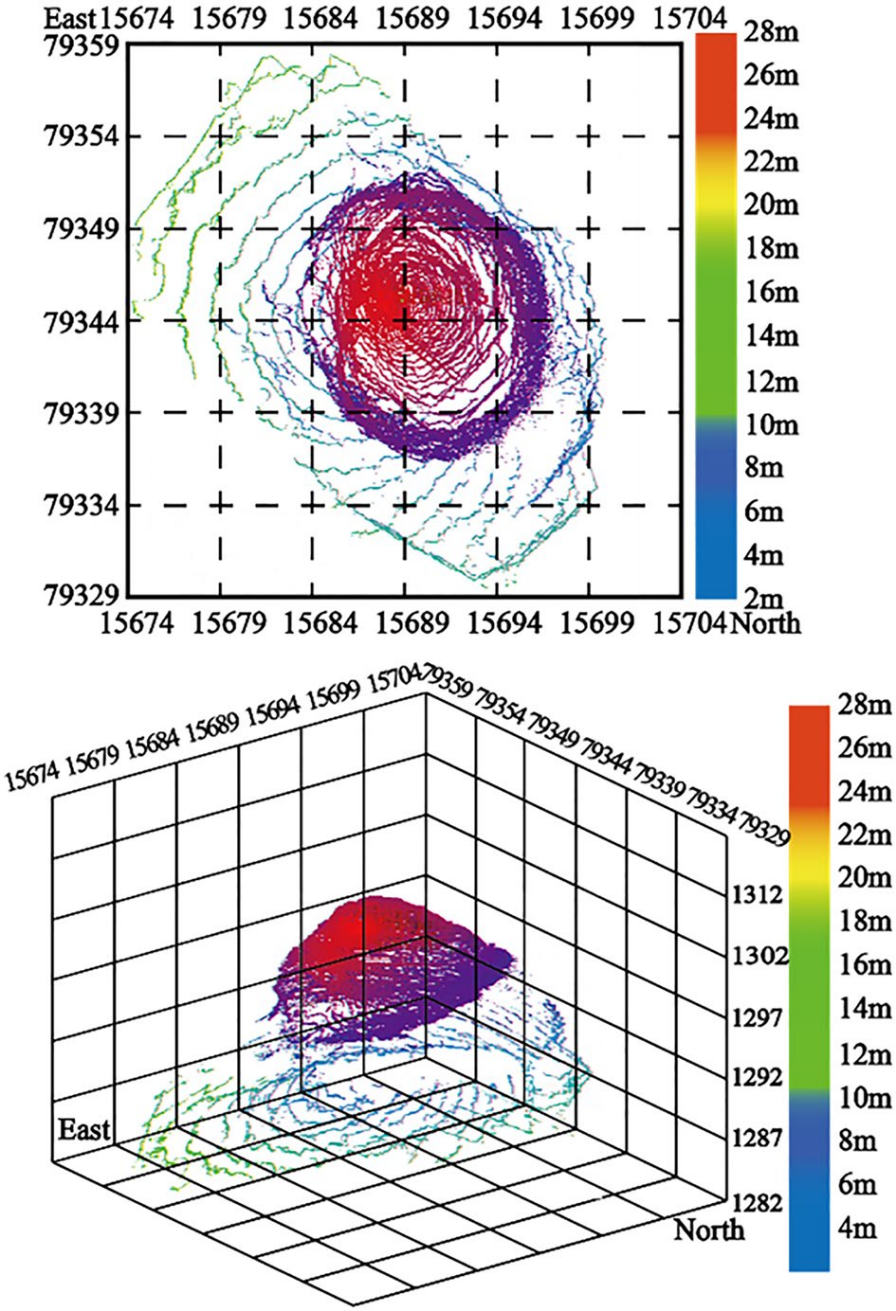
1320 flat plate A-59 drilling 3d laser scanning 3D model. According to the results of three-dimensional laser scanning, the distance between the roof and the floor of the mined-out area is 25.6 m, the cavity volume of the mined-out area is about 3400m^3^, the minimum distance between the roof and the surface is 21 m, the short axis azimuth is 35.7, and the length of the mined-out area.The azimuth angle of the long axis is 129 and the length is about 28.5 m.

According to the scanning results, this kind of mined-out area is mainly distributed in the south-central part of the working gang and the mined-out area of Xinjing Mine at the bottom of the ditch. A typical example is the 1320 flat plate A-59 borehole to expose the mined-out area. According to the results of three-dimensional laser scanning, the distance between the roof and the floor of the mined-out area is 25.6 m, the cavity volume of the mined-out area is about 3400 m^3^, the minimum distance between the roof and the surface is 21 m, the short axis azimuth is 35.7, and the length of the mined-out area.The azimuth angle of the long axis is 129 and the length is about 28.5 m. Arrange the three-dimensional point cloud in the goaf, as shown in Fig. 12.

**Figure 13:**
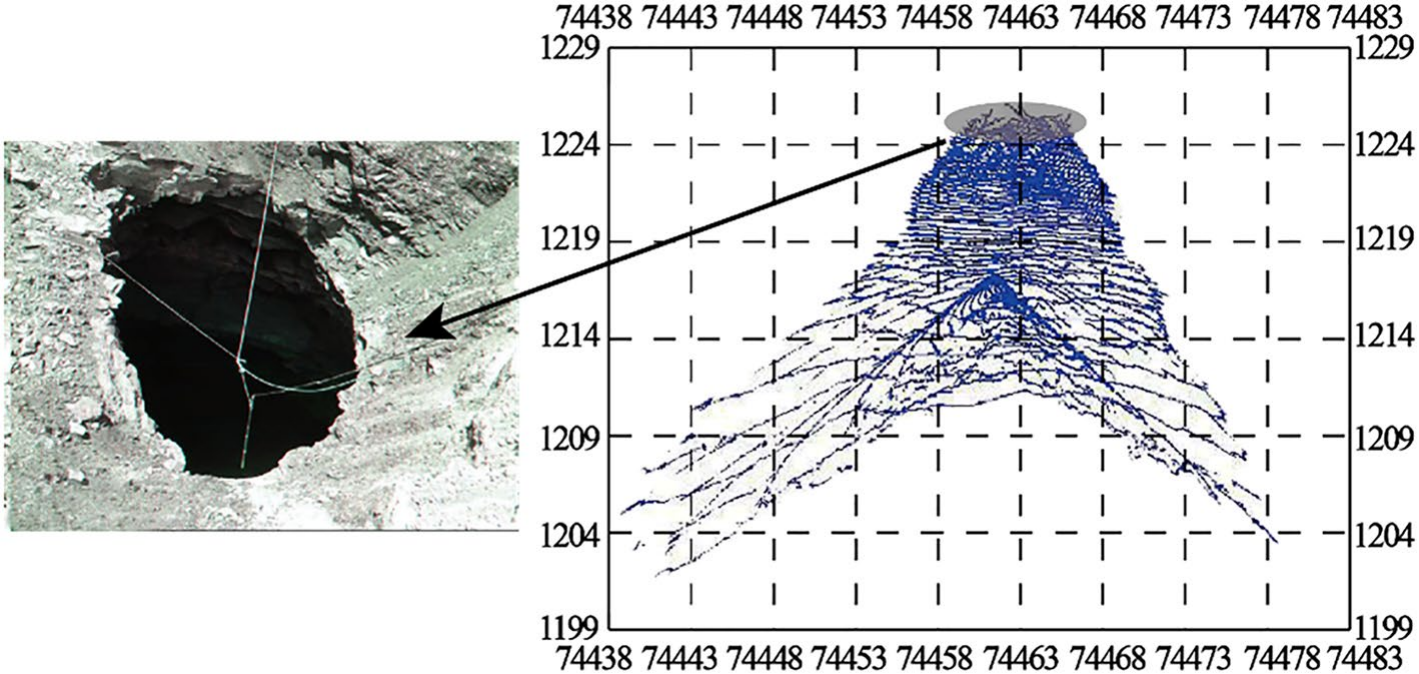
East open-pit mine 1225 flat 0528 cavity. It is also a typical inverted funnel-shaped cavity and this cavity has collapsed to the surface.

**Figure 14:**
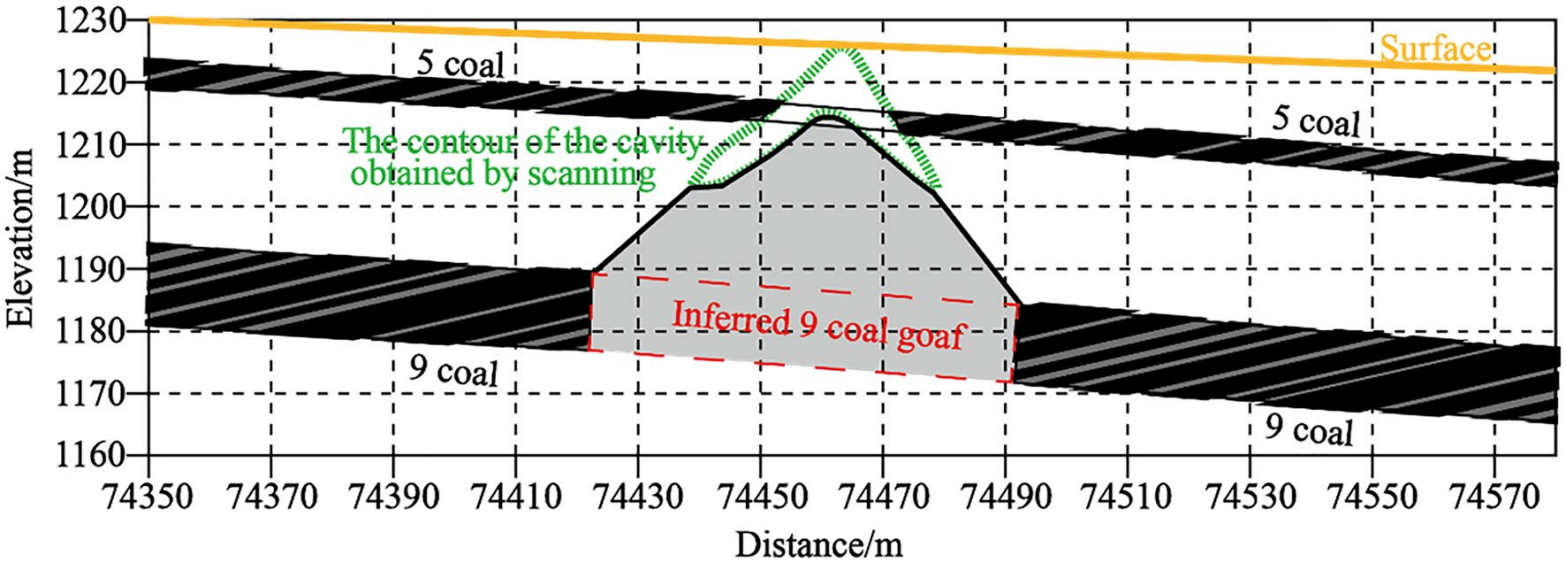
Spatial location of cavity 1225 pingpan 0528 in East open-pit Mine. As shown in the figure, according to the contour of the cavity obtained by scanning, this cavity has collapsed to the surface, the roof of No.9 coal mined-out area collapsed locally and inferred that the north–south span of 9 coal goaf is about 70 m.

The cavity of 1225 flat plate 0528 in East Open-pit Mine is also a typical inverted funnel-shaped cavity. The difference is that this cavity has collapsed to the surface. Due to the long formation time of the mined-out area, the degree of rock weathering is high, and at the same time, due to the combined influence of blasting vibration and transportation load in the open-pit mining process, the roof of No.9 coal mined-out area collapsed locally.It is inferred that the north–south span of 9 coal goaf is about 70 m, as shown in Figs. 13 and 14.

#### Type II

**Figure 15:**
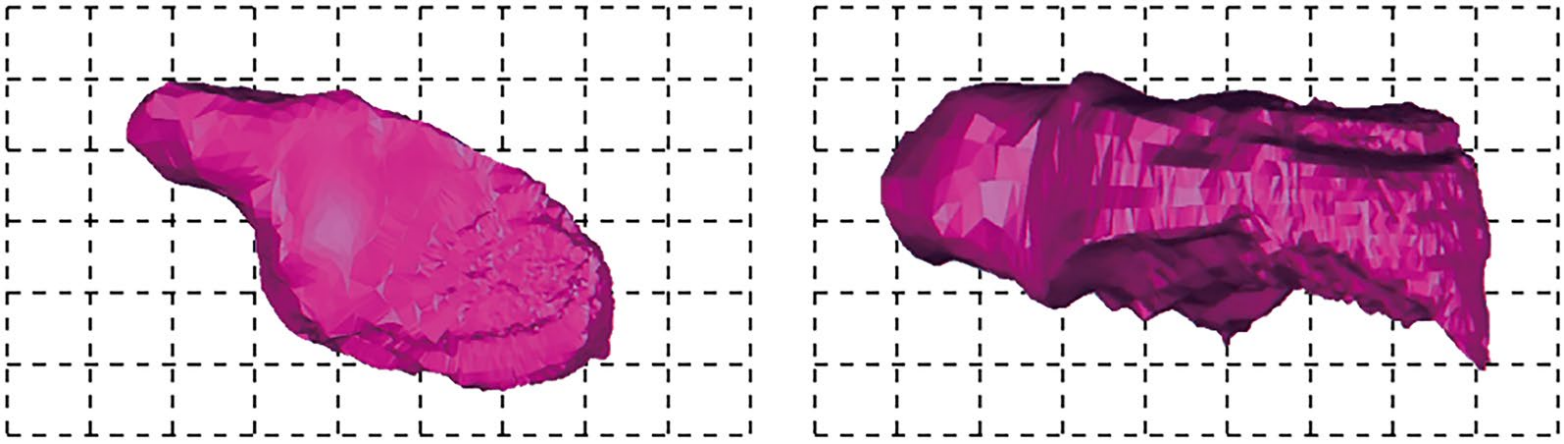
1290 Flat plate 1290-302 Drilling 3D laser scanning 3D model. The roof of the goaf is relatively regular and flush, the thickness of the roof is basically the same.

**Figure 16:**
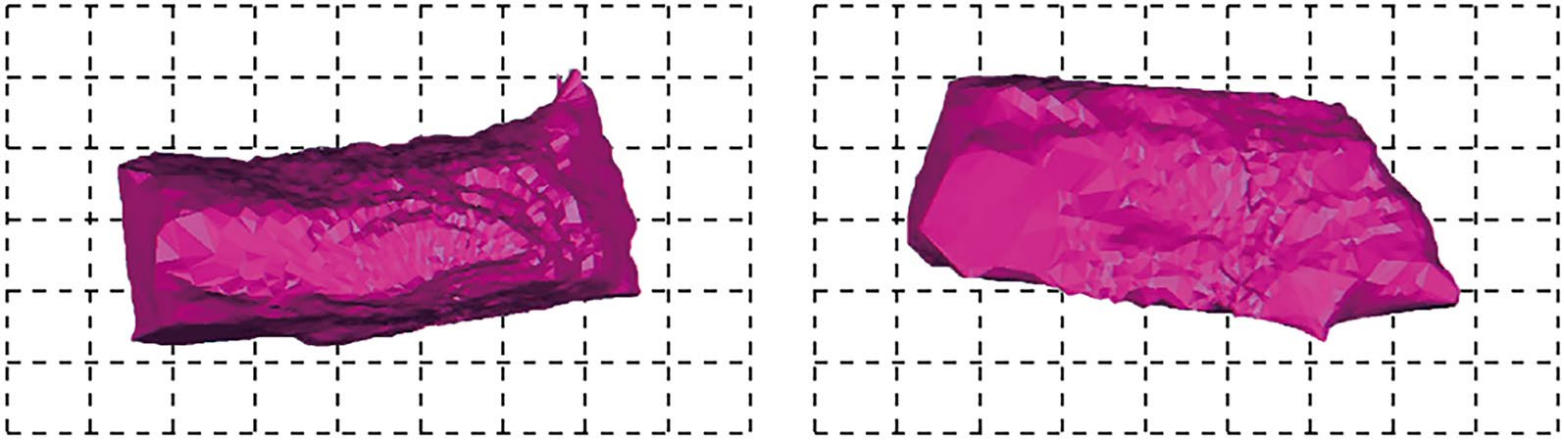
1275 Flat plate 1275-156 Drilling 3D laser scanning 3D model. The roof of the goaf is relatively regular and flush, the thickness of the roof is basically the same.

According to the existing geological data, in the middle of the east open pit, there are sandy rocks with larger thickness and higher hardness on the roof of the coal seam. After the coal seam is mined, the roof of the goaf formed in this area is not easy to collapse, and can still keep its original shape. Therefore, the characteristics of the goaf formed in this area are as follows: the roof of the goaf is relatively regular and flush,The thickness of the roof is basically the same, so it is relatively simple to judge the drilling depth during blasting. The blasting treatment effect of the goaf is largely determined by the blasting effect of the central cut hole. If the blasting effect of the cut hole is not good, it is difficult to form a free space for blasting above the goaf. Further, the collapse of the rock mass above the goaf is incomplete and insufficient, and a "turtle shell" will be formed on the surface, which may eventually lead to the collapse of large equipment here. According to the scanning results, this kind of mined-out areas are mainly distributed in the middle of the working gang. Typical examples are 129 flat plate 1290-302 borehole exposed cavities, 1290 flat plate 1290-303 borehole exposed cavities and 1275 flat plate 1275-156 borehole exposed cavities.As shown in Figs. 15 and 16.

#### Type III

**Figure 17:**
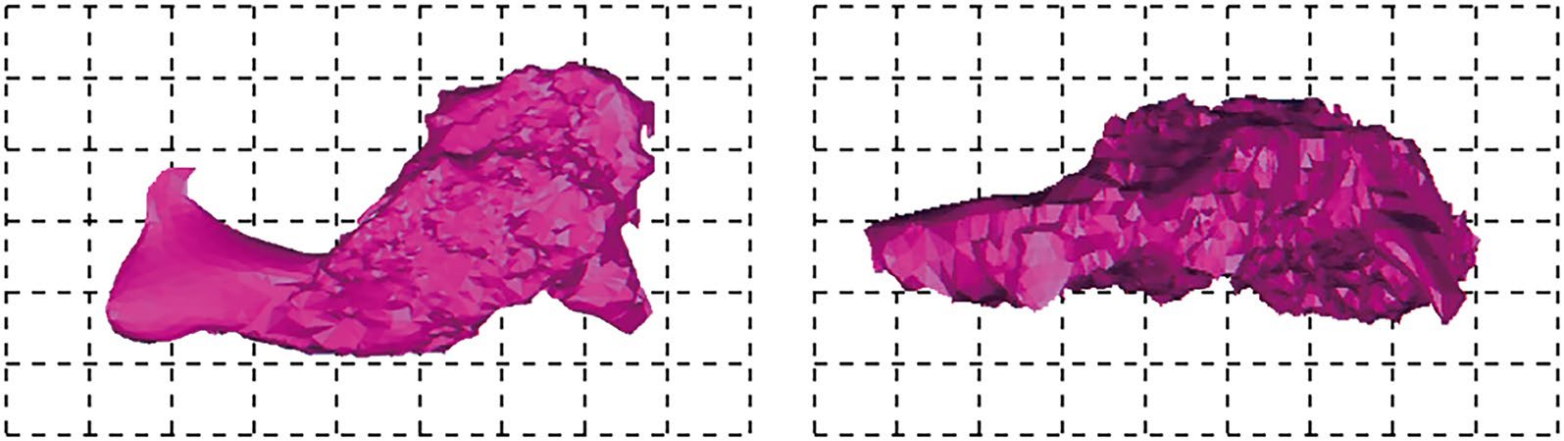
3D model of mined-out area of drilling hole 1320-181, 1320-182 and 1320-183. the geometric shape of mined-out areas can be fully ascertained by joint detection and mutual supplement of multiple holes, this goaf has a large span and the roof caving of goaf is incomplete.

According to the scanning results, it is found that some mined-out areas can't be completely detected by a single scanning hole, and the geometric shape of mined-out areas can be fully ascertained only by joint detection and mutual supplement of multiple holes. Generally, this kind of goaf has a large span, and under the influence of rock weathering and blasting, the roof caving of goaf is incomplete. Goaf treatment is relatively difficult. According to the scanning results, this kind of mined-out areas are mainly distributed in the northwest of the working gang. Typical examples are 1320 flat plates 1320-181, 1320-182 and 1320-183, which are drilled to expose mined-out areas, as shown in Fig. 17.

## Conclusions

In this study, the development and application of geophysical methods related to goaf detection are summarized, in view of the complex geometry of goaf, the surface topography above the mined-out area is generally complex and minimize the disturbance and influence on the overlying rock mass of goaf, the principle of goaf detection scheme formulation is given. Considering the characteristics of many underground small coal mines and many mined-out areas in the East Open-pit Mine, only one exploration method can not accurately obtain the information of mined-out areas, the advantages and disadvantages of different goaf detection methods are compared, the joint detection method of underground goaf is put forward, and the comprehensive exploration scheme of goaf in East Open-pit Mine is formulated. The area, shape, roof thickness and height of the mined-out area are proved. Finally, the hidden mined-out area is visualized by 3D laser scanning technology, it plays an important role in the preparation of subsequent mining plans. the predecessors generally used only one method of geophysical prospecting and each method has its limitations, the joint physical exploration technology can combine the advantages of each exploration technology to detect the complex goaf clearly . The main conclusions are as follows:

1. Aiming at the complex underground goaf groups in East Open-pit Mine, three geophysical methods, SRM, TEM and HRM, are jointly used to detect and identify the goaf. The detection results show that the combined geophysical technology can improve the detection accuracy and overcome the shortcomings and errors caused by single geophysical technology.

2. In the research area with a total area of 3.11 km^2^, 9 inferred mined-out areas were explained by 3D seismic exploration. Transient electromagnetic method is used to delineate 223 abnormal areas at different elevations within the exploration range. 58 drilling holes are arranged in the suspected mined-out area of East Open-pit Mine.The interpretation results of three exploration methods, namely 3D seismic exploration, transient electromagnetic exploration and high-density electrical exploration, have good consistency, and can be verified and complemented with each other, with obvious effect.

3. On the basis of SRM, TEM and HRM detection, borehole exploration and 3D laser scanning are carried out on the mined-out area. Combined with geological software, 3D model map of mined-out area is drawn, and the causes of formation of mined-out area are classified and analyzed.

4. Using 3D laser scanning technology to study the visualization of hidden mined-out areas, the hidden mined-out areas are divided into three types through visualization research, and its formation mechanism is analyzed, which lays a foundation for the stability analysis of hidden mined-out areas.

In summary, the Joint Geophysical Prospecting Technology and 3d laser scanning technology is reliable for clear detection and visualization of East Open-pit Mine. This technology has a certain effect on the detection of open-pit mines which have small underground coal mines and many mined-out areas with complex geometric shapes, therefore, similar open-pit mines are applicable. However, the cost of joint physical exploration technology is generally higher than that of single exploration technology. In some practical projects, may need to strike a balance between cost and efficiency. In the future, the geophysical exploration methods will be continuously enriched and developed, the comprehensive detection and visualize of Hidden Cavity Goaf will have better application prospects.

## Data Availability

The data used to support the findings of this study are available from the corresponding author upon request.
